# LAP-MALDI MS Profiling and Identification of Potential Biomarkers for the Detection of Bovine Tuberculosis

**DOI:** 10.1021/acs.jafc.3c01879

**Published:** 2023-09-07

**Authors:** Sophie E. Lellman, Christopher K. Reynolds, A.K. Barney Jones, Nick Taylor, Rainer Cramer

**Affiliations:** † Department of Chemistry, University of Reading, Whiteknights, Reading RG6 6DX, United Kingdom; ‡ School of Agriculture, Policy and Development, University of Reading, Whiteknights, Reading RG6 6EU, United Kingdom; § Veterinary Epidemiology and Economics Research Unit (VEERU), PAN Livestock Services Ltd, School of Agriculture, Policy and Development, University of Reading, Whiteknights, Reading RG6 6EU, United Kingdom

**Keywords:** bovine tuberculosis, diagnostics, mass spectrometry, matrix-assisted laser desorption/ionization (MALDI), S100-A12

## Abstract

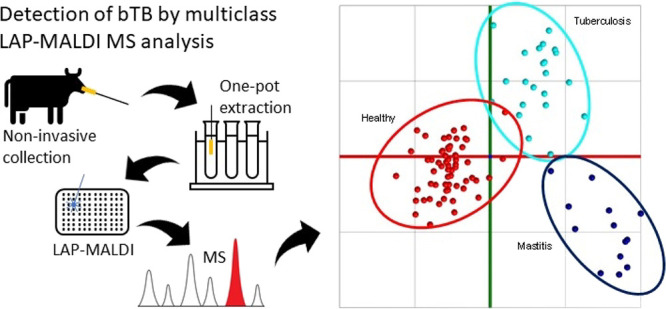

Detecting bovine tuberculosis (bTB) primarily relies on the tuberculin skin test, requiring two separate animal handling events with a period of incubation time (normally 3 days) between them. Here, we present the use of liquid atmospheric pressure (LAP)-MALDI for the identification of bTB infection, employing a three-class prediction model that was obtained by supervised linear discriminant analysis (LDA) and tested with bovine mastitis samples as disease-positive controls. Noninvasive collection of nasal swabs was used to collect samples, which were subsequently subjected to a short (<4 h) sample preparation method. Cross-validation of the three-class LDA model from the processed nasal swabs provided a sensitivity of 75.0% and specificity of 90.1%, with an overall classification accuracy of 85.7%. These values are comparable to those for the skin test, showing that LAP-MALDI MS has the potential to provide an alternative single-visit diagnostic platform that can detect bTB within the same day of sampling.

## 1 Introduction

Bovine tuberculosis (bTB) is a worldwide disease that is devastating for the cattle population and has serious economic and social impacts for dairy farming, with significant risks to the human population through zoonotic transmission.^1^ In Great Britain alone, 3668 new herd incidents were reported between October 2021 and September 2022, with 76% of these being reported in the southwest and west of England, which are deemed high-risk areas by the UK’s Government Department for Environment, Food and Rural Affairs (DEFRA).^2^ In 2013, the UK Government launched various bTB eradication strategies, with the aim of declaring the UK bTB-free by 2038. The main priorities of this program are the development of a cattle vaccine, enforcing wildlife control policies, and improving diagnostic testing.

Overall, bTB costs the UK approximately £100 million per year, with over 27,000 cattle being slaughtered for disease control in 2021.^3^ There are many factors that negatively influence the control of bTB. The causative bacterium, *Mycobacterium bovis*, has a complex life cycle. *M. bovis* can infect humans as well as a wide range of animals, making it difficult to eradicate in British wildlife. In addition, infection with *M. bovis* is usually asymptomatic, with symptoms not presenting until late in disease progression, at the fatal stages of the disease.^3^

In the UK, there are currently two bTB diagnostic tests approved for use. The primary test is the single intradermal comparative cervical tuberculin (SICCT) test, also referred to as the tuberculin skin test, which measures a delayed hypersensitivity reaction in the animal.^4^ Two individual injections of bovine and avian tuberculin are administered under the skin of the animal, and the test is read out 72 h later. If an inflammatory response to bovine tuberculin relative to avian tuberculin is presented on the skin, this is deemed a positive test result. The secondary test is the interferon (IFN)-y blood test, where blood is drawn from the animal and mixed with bovine and avian tuberculin. The levels of cytokine produced in response are subsequently measured. The IFN-y test is used to supplement the tuberculin skin test, particularly in low-risk areas, to detect infections that may not have been detected simply with the skin test. The tuberculin skin test has a high specificity of 99.98%;^5^ however, the sensitivity is only approximately 80%.^6^ It is for this reason that the IFN-y test supplements the tuberculin skin test, with a specificity of 96.6% and sensitivity of 87.6%.^7^

Alternative methods for bTB diagnostics have been investigated to improve the detection rate. Nucleic-acid-based tests such as PCR testing have been used to target the *Mycobacterium tuberculosis* complex, which contains *M. bovis*, providing an average specificity of 97% and sensitivity of 87.7%.^8^ However, the sample collection for this technique is invasive. Typically, the collection of tissue samples from lymph nodes is used, which is not suitable for large-scale diagnostics and imposes additional distress to the animal. Point-of-care antigen tests, which were originally developed for humans, have also been tested against bTB. These have utilized various biological fluids to detect *M. tuberculosis*-specific antigens. However, further research is required on both as both tests have variable efficacies.^9^

MALDI mass spectrometry (MS) has been increasingly used for identification of bacterial infections in human and veterinary diagnostics. For both, the same workflow is employed, whereby a clinical sample is obtained and subsequent culturing is required for the growth/propagation of the pathogenic microorganisms.^10^ However, there have been fewer advances in the rapid, direct analysis of clinical samples for veterinary diagnostics; direct MALDI MS analysis of animal samples is more commonly applied to milk in the context of food adulteration.^11^ The use of LAP-MALDI MS has recently been demonstrated for the detection of bovine mastitis with high specificity and sensitivity.^12^ Only small volumes of milk are required for analysis, and using a quick preparation protocol, lipids, peptides, and proteins can be detected within the mass spectral profile, allowing rapid diagnosis of mastitis 2 days before clinical manifestation.^13^ LAP-MALDI MS contrasts to traditional MALDI MS^14−16^ in that liquid samples are analyzed at atmospheric pressure, as opposed to solid, crystalline samples under a vacuum, allowing simple sample preparation and introduction to the mass analyzer with less interference from matrix-cluster ions as is typically observed in traditional MALDI. It also allows the detection of ESI-like multiply charged ions in a low *m*/*z* range.^17^

In this study, we present a novel application of LAP-MALDI MS profiling in veterinary diagnostics. It is shown that samples from cattle with bovine diseases such as bTB and bovine mastitis can be distinguished from samples of healthy cattle. Bovine samples were collected and prepared using a relatively rapid (limited) digestion method compared to overnight digestion protocols followed by analysis using LAP-MALDI MS. High specificity and sensitivity were obtained for the identification of bTB, mastitis, and healthy bovine samples. This study was funded by the UK government as part of a 25 year initiative to eradicate bTB from the UK by 2038.

## 2 Materials and Methods

### 2.1 Materials

Cotton-tipped wooden swabs, HPLC-grade water, ethanol, acetonitrile, and trifluoroacetic acid (TFA) were purchased from Fisher Scientific (Loughborough, UK).

For the digestion, ammonium bicarbonate (ABC), dithiothreitol (DTT), and iodoacetamide (IAA) were bought from Sigma-Aldrich (Gillingham, UK). Sequencing-grade trypsin was purchased from Promega (Chilworth, UK), and C18 ZipTips for sample clean-up were purchased from Merck (Poole, UK).

For LAP-MALDI matrix preparation, α-cyano-4-hydroxycinnamic acid (CHCA) and propylene glycol were bought from Sigma-Aldrich.

### 2.2 Sample Cohort

Sample collection for this study took place at seven different locations within the UK. Negative control samples were obtained from healthy animals. One of the sites used for the collection of negative controls was at Crichton Royal Farm in Dumfries, Scotland, which has been declared officially bTB-free since 2009. A second collection site for negative control samples was the Centre for Dairy Research (CEDAR) at the University of Reading (Reading, England), which was bTB-free at the time of sampling. The remaining negative controls were collected from farms in West Berkshire (England) on the readout day of tuberculin skin testing. These were sites where positive bTB skin tests were recorded on animals, but samples from these animals were not collected for this study.

As (disease/infection-)positive controls for statistical modeling, nasal swabs were also taken from cows diagnosed with mastitis to determine whether differences are due to a general immune response or are bTB-specific. All mastitis samples were collected from CEDAR. Diagnosis of mastitis was based on the detection of clots present in a quarter of the udder, which was assessed at each milking session (twice daily).

The final class of samples was collected from bTB animals. Five of the bTB samples were obtained from reactor animals from two different farms in West Berkshire. These were collected at the readout stage of the tuberculin skin test. The remaining bTB samples were collected from naturally infected animals being held at the UK’s Animal and Plant Health Agency (APHA) at Weybridge (England). From these animals, a swab was taken from each nostril, totaling two swab samples per animal, except for one animal where only a single sample could be taken.

In total, 60 healthy samples (negative controls), 22 bTB samples (positives), and 13 mastitis samples (disease-positive controls) were collected. Of these, 84 were from female cattle and 11 from male cattle. Details of all samples collected can be found in Supporting Information Table S1.

All reactors were confirmed via postmortem or microbiological culture and were culled after sampling. With the exception of 14 healthy animals without any follow-up health information, all other animals sampled, both healthy and those with mastitis, were otherwise healthy for at least 3 months following sampling (see Supporting Information Table S1).

### 2.3 Sample Collection Procedure

Nasal swabs were used for all sample collections. For each individual sample collection, a swab was inserted into one of the animal’s nostrils for 3–5 s, ensuring that the swab looked wet and was coated with nasal fluid. All swabs were triple-packaged and placed into an ice-filled freezer box for transportation to the laboratory. Upon receipt at the laboratory, samples were placed into a –80 °C freezer for storage. Supporting Information Document S1 provides details of the standard operating procedure (SOP) that was applied for the sample collection.

### 2.4 Sample Preparation for MS Analysis

Once all samples were collected, the samples were removed from the −80 °C freezer for batch processing. Swabs in their casing were immediately transferred into a microbiological safety cabinet, removed from the outer casing, placed into a 1.5 mL tube containing 400 μL of 1× PBS, and briefly agitated at least five times to assist solubilization of biomolecules. All swabs were gently squeezed against the inside walls of the 1.5 mL tube and subsequently discarded. A volume of 900 μL of 100% ethanol was added, and the mixture was vortexed. As multiple samples were processed at the same time, samples were placed on ice at this stage. The sample mixtures were then centrifuged for 5 min at 13,000 rpm. The supernatant was removed and discarded, and the resultant pellet was resuspended in 30 μL of 0.1% TFA.

For the digestion, 50 μL of 50 mM ABC was added to the dissolved sample pellets and mixed by pipetting. For reduction, 5 μL of 100 mM DTT was added to the samples and vortexed followed by incubation at 37 °C for 30 min. For subsequent alkylation, 10 μL of 100 mM IAA was added to the samples and vortexed followed by incubation at room temperature in the dark for 30 min. For the next step of enzymatic digestion, a small volume of 2 μL containing 0.4 μg of trypsin was added, and the samples were incubated at 37 °C for 2 h. The digestion was stopped by acidification with 8 μL of 10% TFA. Samples were then purified using C18 ZipTips according to the manufacturer’s instructions, with a final elution volume of 5 μL of ACN/0.1% TFA (1:1).

A liquid support matrix (LSM) was used for all LAP-MALDI MS measurements. It was formed of CHCA (25 mg/mL) in 70:30 ACN/H_2_O, with PG subsequently added in a ratio of 7:10 (PG/CHCA solution). A volume of 0.5 μL of LSM was spotted onto a stainless-steel MALDI sample plate followed by the addition of 0.5 μL of the freshly prepared sample digest. Supporting Information Document S2 provides details for the SOP that was applied for the sample preparation and subsequent data acquisition and analysis.

### 2.5 MS and MS/MS Data Acquisition

All MS and MS/MS measurements were performed using a Synapt G2-Si (Waters; Wilmslow, UK) with an in-house built AP-MALDI source. Calibration of the instrument was performed using sodium iodide in the *m*/*z* region of 100–2000. A 343 nm diode-pumped solid-state laser was used with a laser pulse repetition rate of 30 Hz and a laser energy of approximately 18 μJ/pulse at the desorption spot. The ion source was operated at 3.0 kV with a counter nitrogen gas flow of 180 L/h and heated capillary. All data acquisition and initial data processing were performed using the MassLynx 4.2 software (Waters). Data acquisition for each sample was for 1 min with one scan per second.

MS/MS data acquisition was performed in mobility TOF mode. Precursor ions were selected, and the quadrupole isolation window was adjusted using LM and HM resolution values, dependent on the precursor ions. Multiple charge states were sequentially selected for fragmentation using collision-induced dissociation (CID). The collision voltage was set at 40 V in the trap cell for the 10+ charge state. Further CID fragmentation spectra were acquired in the transfer cell. The collision voltage varied between 30 and 60 V, depending on the charge state selected for fragmentation.

### 2.6 MS Data Analysis

Statistical analysis of the MS profiles was performed with the Abstract Model Builder (AMX; [Beta] Version 1.0.1962.0; Waters). All data files were imported to the AMX software, and spectra from all scans per file were selected. For all data files, binning of mass spectral data was performed every unit value in the *m*/*z* range of 700–1800. Linear discriminant analysis (LDA) was selected for all analyses with a preprocessing method using principal component analysis (PCA) for dimensionality reduction. Following PCA, LDA was applied to determine the maximum variation between the applied classes of sample (“Healthy”, “bTB”, and “Mastitis” or simply “Healthy” and “Diseased”). Cross-validation of the LDA models was performed using the built-in “20% out” function. For PCA, 50 dimensions were chosen, whereas for LDA, the number of dimensions was 1 and 2 for the two- and three-class analysis, respectively. Outliers were defined by 5 standard deviations.

### 2.7 MS/MS Data Analysis

Ion mobility filtering was applied postacquisition to remove interfering singly charged ions from the mass spectrum. A band selection was applied, and the data were exported to MassLynx, retaining the drift time.

As all data were acquired in mobility TOF mode, the fragment ion peak list was created manually (due to file compatibility reasons) and searched using MS/MS Ion Search of the MASCOT search software (version 2.7; Matrix Science; London, UK). For the identification of larger proteins, only the singly charged fragment ions that were common to more than one fragmentation spectrum were included in the peak list. All multiply charged fragment ions of each fragmentation spectrum obtained from the different multiple charge states were also included in the peak list as [M + H]^+^ ions. To obtain the mass values for the [M + H]^+^ ions, the multiply charged fragment ion signals were deconvoluted using the MaxEnt plug-in for MassLynx, with a deconvoluted molecular mass range of 100–11,000 Da and a maximum of 10 charges. Deconvoluted signals of the fragment ions obtained by MaxEnt were verified by checking the actual MS/MS spectra for their appearances. Fragment ion peak lists were searched against all taxonomies using the NCBIprot database (version 20201010) with ±75 ppm peptide mass tolerance and ±0.2 Da fragment mass tolerance. Larger proteins were searched with “NoCleave” as the enzyme, and presumed tryptic peptides were searched using trypsin as the enzyme, allowing for one missed cleavage.

## 3 Results

Because of varying collection dates, all swabs were stored at −80 °C in quarantine until all swabs had been collected to process all samples at the same time. When all samples were collected, the swabs underwent a simple precipitation procedure using ethanol to concentrate biomolecules within the sample as well as for the inactivation of *M. bovis* and any other hazardous microorganisms that may be present for health and safety purposes. Samples were spun down, and the pellets were resuspended in 0.1% TFA. In preliminary testing, the analysis of the resolubilized pellets did not yield informative results, and therefore, a short enzymatic digestion step was added. The use of LAP-MALDI MS is somewhat limited in the detection of larger biomolecules. Hence, a digestion step was added to cleave any larger proteins into smaller fragments, facilitating their detection by LAP-MALDI MS.

All LAP-MALDI samples were spotted onto a 96-well MALDI sample plate and analyzed sequentially by acquiring MS profiles in the *m*/*z* range of 100–2000. Following the acquisition of the MS profile data, the data files were imported into the AMX model builder for statistical analysis. The *m*/*z* region below 700 was not included in the data analysis for class modeling as there are only a few analytes of interest in this region, whereas ion signals from the MALDI matrix and contaminations can be present to a greater extent, thus limiting the influence of nonspecific ion signals on the data modeling.

**Figure 1:**
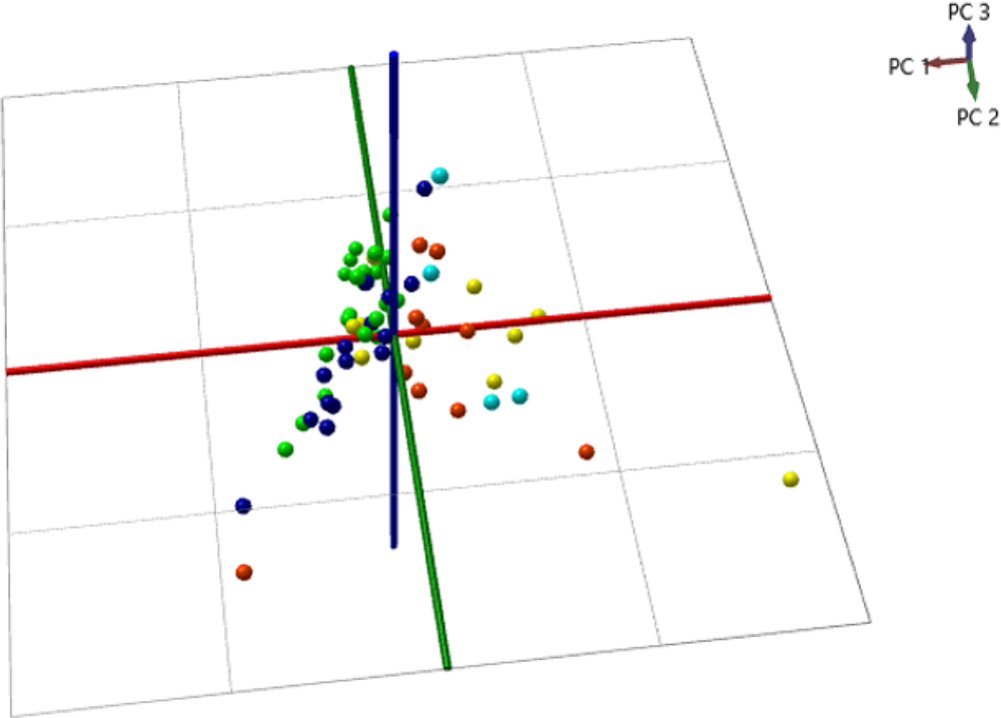
Visualization of PCA of samples from healthy animals to determine any bias due to their geographical location. Each geographical location is represented by a different color.

Both LDA and PCA were applied to the obtained MS profile data set. LDA is a supervised technique, taking into account the assigned classes prior to building a classification model, maximizing the difference between the classes. PCA is an unsupervised technique, maximizing variation in the whole data set without using any known class labels. PCA was used in this study to evaluate whether any principal component could be found that can easily cluster the profile data according to other variables than the health status, in particular, the geographic location of the animals. For this, only the profiles of healthy animals were interrogated. Figure 1 shows a visualization of the obtained PCA data, whereby through cross-validation all samples were classified as outliers.

**Figure 2:**
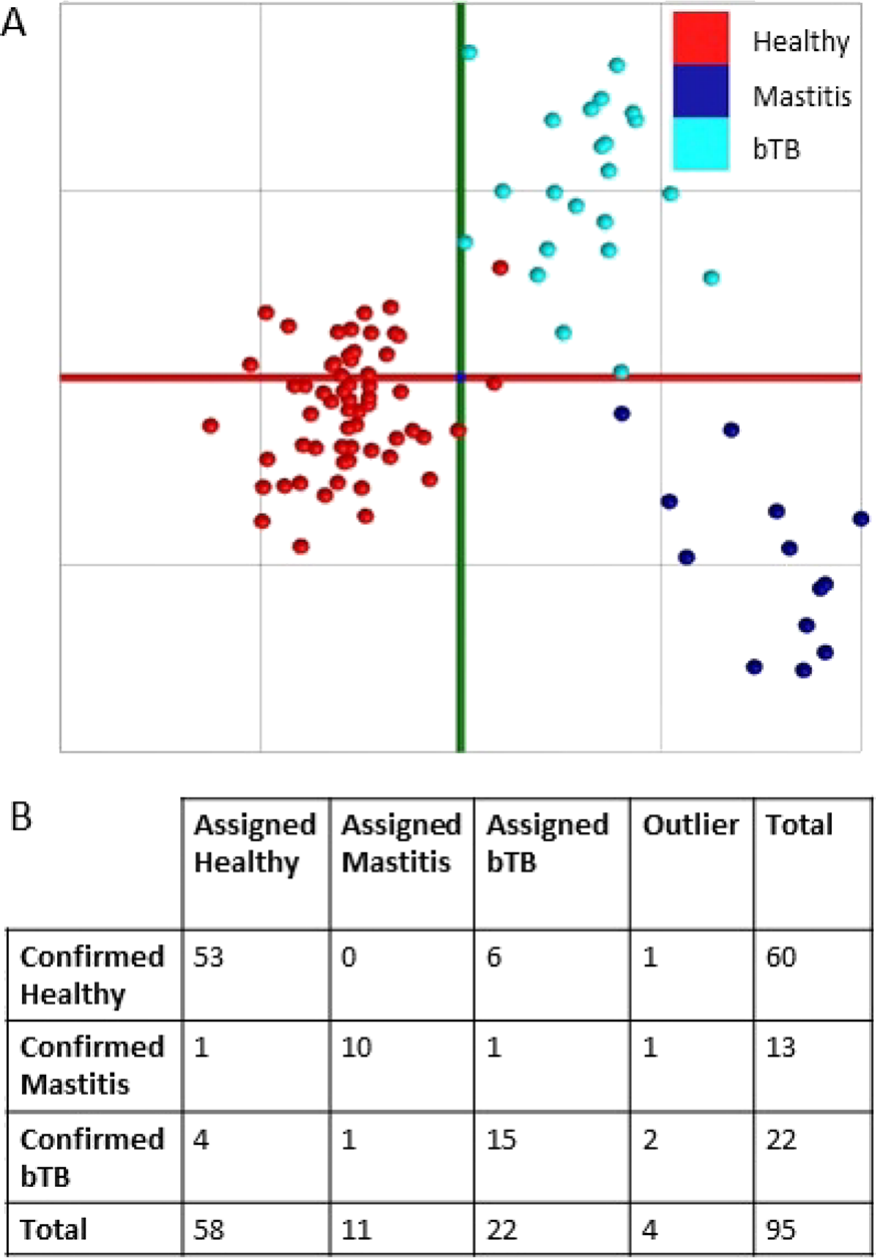
(A) Visualization of the linear discriminant analysis (LDA) for discrimination between healthy, mastitis, and bTB samples. (B) Confusion matrix detailing the assignments based upon the LDA model in panel A using “20% out” cross-validation.

Using the entire profile data set (limited to the *m*/*z* region of 700–1800), LDA was used for building prediction models to classify healthy (negative controls), mastitis (positive controls), and bTB animals. Figure 2 presents the obtained LDA classification data, demonstrating an overall classification accuracy of 85.7% with a bTB detection sensitivity of 75.0% and specificity of 90.1% when applying the “20% out” cross-validation (excluding four outliers, whose analysis would be repeated in routine testing). PCA was also performed on this data set, leading to no outliers but providing a lower cross-validation accuracy of 72% compared to LDA.

**Figure 3:**
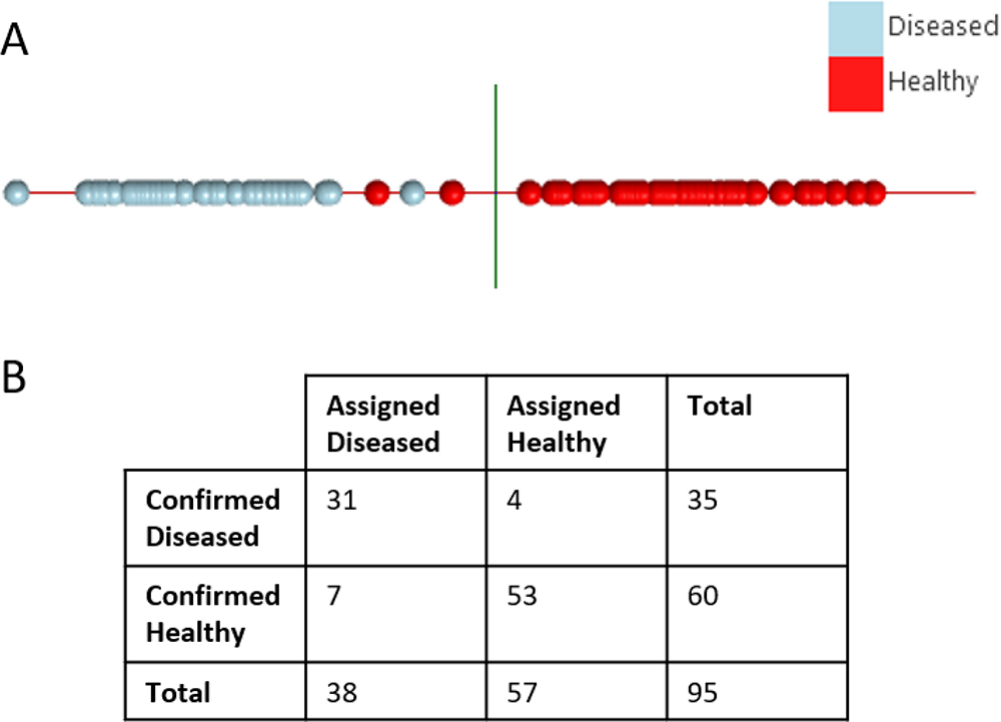
(A) Visualization of the linear discriminant analysis (LDA) for the discrimination between samples from healthy and samples from diseased (bTB and mastitis) animals. (B) Confusion matrix detailing the identification of samples from healthy and diseased animals based upon the LDA model in panel A using “20% out” cross-validation.

The class labels were then simplified to healthy (negative control) and diseased (mastitis or bTB). For this two-class system, the cross-validated LDA model provided an overall classification accuracy of 88.4% with a sensitivity of 88.6% and specificity of 88.3% (Figure 3).

**Figure 4:**
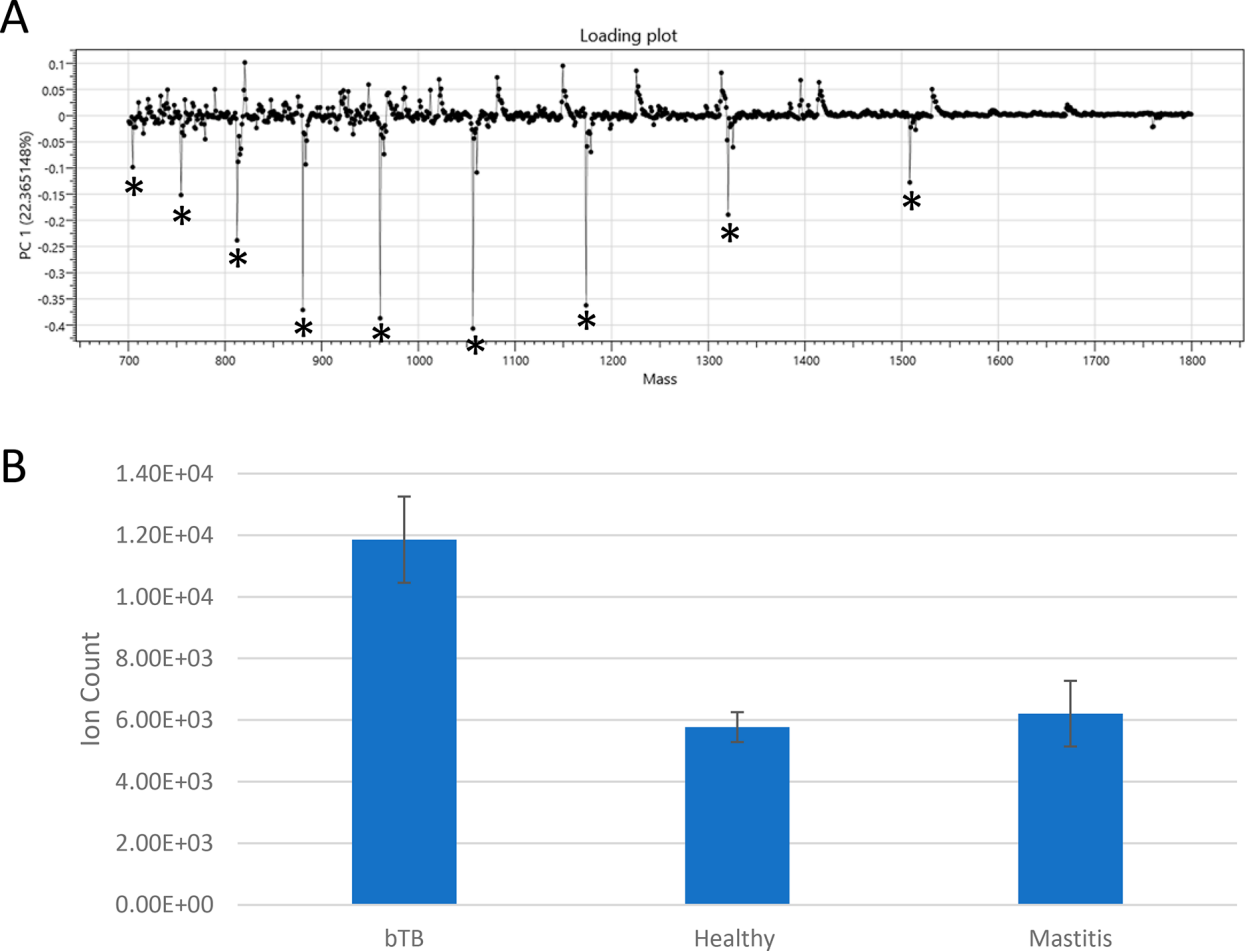
(A) Loading plot for the first principal component of the principal component analysis (PCA), indicating the peaks most responsible for discrimination. Peaks labeled with an asterisk belong to the ions of S100-A12 as identified by MS/MS analysis of the [M + 10H]^10+^ ions at the *m*/*z* value of approximately 1056. (B) Relative mean ion signal intensities of the overall strongest S100-A12 ion signal ([M + 12H]^12+^; *m*/*z* 880) for each sample class. The error bars provide the standard errors.

From the loading plot of the PCA dimensionality reduction (Figure 4A), one putative biomarker protein was identified as being highly responsible for the variation in the data set. The related ion signals were easily identified in the LAP-MALDI MS profiles (see Supporting Information Figure S1). The protein’s [M + 10H]^10+^ ions were fragmented by CID and analyzed using top-down LAP-MALDI MS/MS as described in Section 2.7. The fragmentation spectrum of the [M + 10H]^10+^ ions can be found in Supporting Information Figure S2, and the fragment ion peak list used for database searching can be found in Supporting Information Document S3. The obtained MS/MS data were searched against the NCBIprot protein database as described in Section 2.7, allowing for all taxonomies. The only significant hits obtained from this search were for bovine proteoforms of S100-A12 (NCBIprot accession number NP_777076), which is a protein that is released by inflammatory cells in response to environmental cues. S100-A12 was identified with an ion score of 43, where individual ion scores >22 indicate significant homology and scores >42 indicate identity. Searching the same data against the SwissProt (version 2023_03) protein database also led to the identification of S100-A12 (P79105) with the same ion score. Figure 4B shows the relative ion signal intensities of S100-A12 for the individual sample classes.

## 4 Discussion

Current bTB testing typically employs methods that are invasive such as the tuberculin skin test and the IFN-y blood test. Both tests are invasive and take time, typically 72 h for the tuberculin skin test, requiring two farm visits, reagents, and consumables that add to the overall costs of the test. Thus, less invasive and faster tests, while being more cost-effective, would improve bTB detection and disease management.

Earlier studies analyzing noninvasively collected milk from dairy cows showed that a simple one-pot sample preparation without any disease-specific reagents is all that is needed for preparing samples to detect mastitis by LAP-MALDI MS profiling.^12^ Further method development and application to a larger, longitudinally collected sample set of bovine milk demonstrated that LAP-MALDI MS profiling was able to detect mastitis up to 2 days before its clinical detection. The cost for large-scale application based on daily sampling of large herds was calculated to be less than US $0.1 per sample.^13^

In this present study, a similar analytical approach was employed by utilizing LAP-MALDI MS profiling. However, no blood or milk but the nasal fluids from the cattle’s nostrils were collected, being less invasive than current bTB tests and allowing disease detection for male animals. Compared to the analysis of milk by LAP-MALDI MS, a wooden swab was used to collect the sample, and a short, limited proteolysis step was added to the sample preparation. The latter required no disease-specific reagents and added only marginally to the costs of consumables when compared to current bTB tests. After the collected samples reach the analytical laboratory, the total sample preparation and analysis time (to result) can be as short as 4 h, substantially faster than the tuberculin skin test. The LAP-MALDI MS platform is also capable of high-throughput analysis,^18,19^ and large-scale population screening is therefore a possibility.

Although the data obtained so far are still limited by the number of bTB animals and overall sample numbers, they clearly demonstrate the potential of LAP-MALDI MS for disease classification beyond the detection of mastitis from fluids that are not as rich as milk with respect to disease-specific biomarkers. For the three-class model, the sample set provided an overall classification accuracy of 85.7%, with a bTB detection sensitivity and specificity of 75.0 and 90.1%, respectively. For simply classifying healthy vs diseased, the accuracy was 88.4%, with a sensitivity of 88.6% and specificity of 88.3%. A review of bTB testing methods showed that most of the reviewed tuberculin skin test studies had an extremely high specificity of typically 90–100% but a much lower sensitivity, being therefore described as “imperfect”.^20^ The sensitivity and specificity values obtained with LAP-MALDI MS profiling are similar but with the potential to further improve once the prediction model has been refined by a larger data set.

In this context, it should be noted that the outliers obtained by the three-class LDA model are based upon 5 standard deviations and were excluded from the final percentage values that are presented within this article. In “real-life” testing, samples classified as outliers would be reanalyzed. Only in the case that a sample is still classified as an outlier would the animal have to be swabbed again.

The loading plot of the PCA dimensionality reduction (Figure 4A) shows that one protein has a large influence on the variation in principal component 1, which accounts for 22.39% of the variation. Loading plots are typically used in unsupervised statistical analysis to reveal the peaks most responsible for the variation in the data set. With the AMX software, the data are initially linearized into principal components followed by the application of the class labels and subsequent LDA. A loading plot can then be viewed for the underlying principal components used in the LDA. From this loading plot, further MS analysis, and subsequent LAP-MALDI MS/MS analysis, protein S100-A12 was identified as a key protein responsible for the discrimination between healthy and the two diseases, with the difference between bTB and healthy being the greatest (Figure 4B).

Despite the applied (though limited) digestion step, S100-A12 was identified in the MS profile as the full-length protein with the N-terminal (initiator) methionine removed. After the digestion step, there were many doubly charged peptides in the LAP-MALDI mass spectrum, which suggest that the digestion was successful for other proteins that were in the sample. Some of these were identified as tryptic peptides from various sources, including bovine IgA (NCBIprot accession number G3MXB5) and rape seed storage protein (NCBIprot accession number CDY29281.1). That S100-A12 was detected as a virtually intact protein despite the digestion step can be explained by its known resistance to protease digestion,^21^ with many of the lysine residues being located next to aspartic acid residues. Trypsin digestions can take longer when lysine and arginine residues are located next to acidic amino acids.^22^ Interestingly, it was not possible to detect the protein without the limited digestion step.

S100-A12 is known to bind to a receptor for advanced glycation end-products (RAGE), whose activation leads to proinflammatory effects.^21^ In humans, S100-A12 is implicated in many diseases, including coronary heart disease,^23^ periodontitis,^24^ as well as lung disease, including pulmonary tuberculosis.^25^ S100-A12 has been previously detected in bovine milk and described as a marker of subclinical mastitis.^26^ It has also been reported in cows infected with *Mycobacterium avium* ssp. *paratuberculosis.*(27) The importance of S100-A12 in response to many diseases, including infectious diseases such as bovine mastitis, and infections with *M. avium* is in good agreement with its identification as a disease marker in this study.

The aim of this study was to evaluate and adopt the LAP-MALDI MS platform for its application to bTB detection. In comparison to current first-line testing using the tuberculin skin test, the results of this study showed a similar sensitivity and specificity with a much faster procedure and a less invasive farm-site sample collection. The acquisition of nasal swabs can be performed by trained veterinary or farm staff while cattle are safely restrained within a cattle crush (squeeze chute). The nasal fluid collection is fast, and the required sample volumes are low (<0.5 mL).

Given the proof-of-principle nature of this study and the limited sample set, further improvements can be expected. To develop this method further, a wider and larger sample set is desirable, with a greater variety of breeds, geographical locations, and other diseases. In particular, diseases that are closely related to bTB such as Johne’s disease, bovine pneumonia, or other respiratory diseases should be used as disease-positive controls. These diseases are very similar in their clinical presentation and can often interfere with current bTB diagnostics. Batch-to-batch variation should also be assessed for clinical use. However, LAP-MALDI MS profiling and subsequent LAP-MALDI MS/MS analyses from the same samples used for profiling have demonstrated that this approach is based on the detection of disease-relevant biomarkers such as S100-A12. These and others from further combined LAP-MALDI MS profiling and MS/MS analyses could ultimately provide individual biomarkers or panels of biomarkers that are highly disease-specific, which could also be exploited for lateral flow antigen tests. The use of LAP-MALDI MS often also removes the need for lengthy chromatography steps that are commonly used with ESI MS(/MS) analysis.

The ability to distinguish between healthy cattle as well as two disease states shows the potential for this platform to be used in multiplex analyses, making it highly versatile and even more cost-effective. Because of its simplicity, speed, and low consumable costs, there is also the potential for large-scale population screening. Similar to the tuberculin skin test, LAP-MALDI MS profiling could be employed in first-line testing followed by further testing modalities as is currently the case with bTB testing. Its speed, i.e., faster readout, might also make it an attractive proposition for earlier intervention and disease management even if the test accuracy cannot be further improved or will be ultimately lower than for the tuberculin skin test.

## Data Availability

Data supporting the results reported in this paper are openly available from the University of Reading Research Data Archive at https://doi.org/10.17864/1947.000443.

## Supporting Information Available

List of animals and their follow-up health status (Table S1; SOP for nasal swab sample collection (Document S1); SOP for nasal swab analysis by LAP-MALDI MS (Document S2); LAP-MALDI mass spectrum of a bTB sample (Figure S1); fragmentation spectrum of the [M + 10H]^10+^ signal of S100-A12 (Figure S2); and peak list used for identifying S100-A12 (Document S3) (PDF)
