# Supplementary material for: LAP-MALDI MS Profiling and Identification of Potential Biomarkers for the Detection of Bovine Tuberculosis
Source: J Agric Food Chem. 2023 Sep 7;71(37):13899–905. doi: 10.1021/acs.jafc.3c01879 (PMC10515614; doi:10.1021/acs.jafc.3c01879)
Supplement: Supplementary file 1 — jf3c01879_si_001.pdf [file jf3c01879_si_001.pdf]

Supporting Information

LAP-MALDI MS profiling and identification of potential biomarkers for the detection of bovine tuberculosis

Sophie E. Lellman[1], Christopher K. Reynolds, A. K. Barney Jones, Nick Taylor and Rainer Cramer[1]*

[1]Department of Chemistry, University of Reading, Whiteknights, Reading, RG6 6DX, United Kingdom

[2]School of Agriculture, Policy and Development, University of Reading, Whiteknights, Reading RG6 6EU, United Kingdom

[3]Veterinary Epidemiology and Economics Research Unit (VEERU), PAN Livestock Services Ltd, School of Agriculture, Policy and Development, University of Reading, Whiteknights, Reading RG6 6EU, United Kingdom

*Address correspondence to:

Prof. Rainer Cramer, Department of Chemistry, University of Reading, Whiteknights, Reading RG6 6DX, UK.

Tel.: +44-118-378-4550

*Email: r.k.cramer@reading.ac.uk

Contents

Table S1 – List of Animals and their Follow-up Health Status

Page 3

Document S1 – SOP for Nasal Swab Sample Collection

Page 6

Document S2 – SOP for Nasal Swab Analysis by LAP-MALDI MS

Page 11

Figure S1 – LAP-MALDI Mass Spectrum of a bTB Sample

Page 17

Figure S2 – Fragmentation Spectrum of the $[M+10H]^{10+}$ Signal of S100-A12

Page 18

Document S3 – Peak List Used for Identifying S100-A12

Page 19

Table S1 – List of Animals and their Follow-up Health Status

Source	Animal ID	Sample Type	Sample Code	Sex	Breed	Follow-Up Health Status
APHA	C21-2488L	Reactor	APHAC21-2488L	Female	Holstein	Culled
APHA	C21-2488R	Reactor	APHAC21-2488R	Female	Holstein	Culled
APHA	C21-2485L	Reactor	APHAC21-2485L	Female	Holstein	Culled
APHA	C21-2485R	Reactor	APHAC21-2485R	Female	Holstein	Culled
APHA	C21-2487L	Reactor	APHAC21-2487L	Female	Holstein	Culled
APHA	C21-2487R	Reactor	APHAC21-2487R	Female	Holstein	Culled
APHA	C21-2486L	Reactor	APHAC21-2486L	Female	Holstein	Culled
APHA	C21-2486R	Reactor	APHAC21-2486R	Female	Holstein	Culled
APHA	2559L	Reactor	APHA2559L	Female	Holstein	Culled
APHA	2560L	Reactor	APHA2560L	Female	Holstein	Culled
APHA	2560R	Reactor	APHA2560R	Female	Holstein	Culled
APHA	2561L	Reactor	APHA2561L	Male	Holstein	Culled
APHA	2561R	Reactor	APHA2561R	Male	Holstein	Culled
APHA	2562L	Reactor	APHA2562L	Female	Holstein	Culled
APHA	2562R	Reactor	APHA2562R	Female	Holstein	Culled
APHA	2563L	Reactor	APHA2563L	Female	Holstein	Culled
APHA	2563R	Reactor	APHA2563R	Female	Holstein	Culled
Farm 021140001	1	Control	02114001C1	Female	Holstein	Unknown
Farm 021140001	2	Control	02114991C2	Female	Holstein	Unknown
Farm 021140001	3	Control	02114991C3	Female	Holstein	Unknown
Farm 021140001	4	Control	02114991C4	Female	Holstein	Unknown
Farm 021140001	50	Reactor	02114991R050	Female	Holstein	Culled
Farm 021140001	343	Reactor	02114991R343	Female	Holstein	Culled
Farm 021140001	3240	Reactor	02114991R3240	Female	Holstein	Culled
Farm 021140001	2637	Reactor	02114991R2637	Female	Holstein	Culled
Farm F	307406	Reactor	F-R307406	Female	Holstein	Culled
Farm K	400614	Control	KFC400614	Female	Charolais	Healthy
Farm K	300963	Control	KFC300963	Female	Charolais	Healthy
Farm K	200969	Control	KFC200969	Female	Charolais	Healthy
Farm K	400901	Control	KFC400901	Female	Charolais	Healthy
Farm K	600973	Control	KFC600973	Female	Charolais	Healthy
Farm K	700981	Control	KFC700981	Female	Charolais	Healthy
Farm K	300956	Control	KFC300956	Female	Charolais	Healthy
Farm K	300935	Control	KFC300935	Female	Charolais	Healthy
Farm K	00090	Control	KFC00090	Female	Charolais	Healthy
Farm K	600504	Control	KFC600504	Female	Charolais	Healthy

Farm U	01	Control	UKNC01	Female		Unknown
Farm U	02	Control	UKNC02	Female		Unknown
Farm U	03	Control	UKNC03	Female		Unknown
Farm U	04	Control	UKNC04	Female		Unknown
Farm U	05	Control	UKNC05	Female		Unknown
Farm U	06	Control	UKNC06	Female		Unknown
Farm U	07	Control	UKNC07	Female		Unknown
Farm U	08	Control	UKNC08	Female		Unknown
Farm U	09	Control	UKNC09	Female		Unknown
Farm U	10	Control	UKNC10	Female		Unknown
CEDAR	7037	Control	CEDAR7037	Male	Angus	Healthy
CEDAR	7035	Control	CEDAR7035	Female	Angus	Healthy
CEDAR	7047	Control	CEDAR7047	Male	Angus	Healthy
CEDAR	7034	Control	CEDAR7034	Female	Angus	Healthy
CEDAR	7038	Control	CEDAR7038	Male	Angus	Healthy
CEDAR	7063	Control	CEDAR7063	Female	Angus	Healthy
CEDAR	7039	Control	CEDAR7039	Female	Angus	Healthy
CEDAR	7076	Control	CEDAR7076	Male	Angus	Healthy
CEDAR	7033	Control	CEDAR7033	Male	Angus	Healthy
CEDAR	7059	Control	CEDAR7059	Male	Angus	Healthy
CEDAR	7016	Control	CEDAR7016	Female	Angus	Healthy
CEDAR	7069	Control	CEDAR7069	Female	Angus	Healthy
CEDAR	7036	Control	CEDAR7036	Male	Angus	Healthy
CEDAR	7050	Control	CEDAR7050	Male	Angus	Healthy
CEDAR	7019	Control	CEDAR7019	Male	Angus	Healthy
CEDAR	2833	Mastitis	CEDARM2833	Female	Holstein	Healthy
CEDAR	2738	Mastitis	CEDARM2738	Female	Holstein	Healthy
CEDAR	3013	Mastitis	CEDARM3013	Female	Holstein	Healthy
CEDAR	2901	Mastitis	CEDARM2901	Female	Holstein	Healthy
CEDAR	3257	Mastitis	CEDARM3257	Female	Holstein	Healthy
CEDAR	2182	Mastitis	CEDARM2182	Female	Holstein	Healthy
CEDAR	2543	Mastitis	CEDARM2543	Female	Holstein	Healthy
CEDAR	2519	Mastitis	CEDARM2519	Female	Holstein	Healthy
CEDAR	3001	Mastitis	CEDARM3001	Female	Holstein	Healthy
CEDAR	2678	Mastitis	CEDARM2678	Female	Holstein	Healthy
CEDAR	2229	Mastitis	CEDARM2229	Female	Holstein	Healthy
CEDAR	3099	Mastitis	CEDARM3099	Female	Holstein	Healthy
CEDAR	2779	Mastitis	CEDARM2779	Female	Holstein	Healthy
Crichton Royal	259	Control	CRC259	Female	Holstein Freesian	Healthy
Crichton Royal	66	Control	CRC66	Female	Holstein Freesian	Healthy
Crichton Royal	505	Control	CRC505	Female	Holstein Freesian	Healthy
Crichton Royal	138	Control	CRC138	Female	Holstein Freesian	Healthy

Crichton Royal	198	Control	CRC198	Female	Holstein Freesian	Healthy
Crichton Royal	523	Control	CRC523	Female	Holstein Freesian	Healthy
Crichton Royal	169	Control	CRC169	Female	Holstein Freesian	Healthy
Crichton Royal	219	Control	CRC219	Female	Holstein Freesian	Healthy
Crichton Royal	595	Control	CRC595	Female	Holstein Freesian	Healthy
Crichton Royal	483	Control	CRC483	Female	Holstein Freesian	Healthy
Crichton Royal	2587	Control	CRC2587	Female	Holstein Freesian	Healthy
Crichton Royal	2822	Control	CRC2822	Female	Holstein Freesian	Healthy
Crichton Royal	577	Control	CRC577	Female	Holstein Freesian	Healthy
Crichton Royal	100	Control	CRC100	Female	Holstein Freesian	Healthy
Crichton Royal	511	Control	CRC511	Female	Holstein Freesian	Healthy
Crichton Royal	2894	Control	CRC2894	Female	Holstein Freesian	Healthy
Crichton Royal	207	Control	CRC207	Female	Holstein Freesian	Healthy
Crichton Royal	591	Control	CRC591	Female	Holstein Freesian	Healthy
Crichton Royal	559	Control	CRC559	Female	Holstein Freesian	Healthy
Crichton Royal	476	Control	CRC476	Female	Holstein Freesian	Healthy
Crichton Royal	200	Control	CRC200	Female	Holstein Freesian	Healthy

Document S1 – SOP for Nasal Swab Sample Collection

Standard Operating Procedure (SOP) for oral and nasal swab collection for the diagnosis of bovine tuberculosis (bTB)

Version 1.3 (24th November 2021)

Barney Jones, Sophie Lellman and Rainer Cramer
Department of Chemistry, UoR

Contents

1. Purpose 8
2. Scope 8
3. Health and Safety 8
4. Recommended equipment/material and personnel required (per collection event) 9
   4.1 Equipment 9
   4.2 Disposables 9
   4.3 Personnel required 9
5. Procedural guidelines 9
   5.1 Sample Collection Procedure 9
   5.2 Packaging for Transport 10
   5.3 Receipt of Samples in the Laboratory 10

1. Purpose

The purpose of this document is to provide guidance for sample collection of oral and nasal swabs from cattle for the further downstream processing and analysis by mass spectrometry.

2. Scope

This SOP is designed for the appropriate collection of nasal and oral swabs from cattle for disease diagnostics. Sample collection is performed by trained research, farm or veterinary staff, in many cases at the time of reading the tuberculin skin test. In order to efficiently detect biomarkers for indicators of disease it is essential that a robust sample preparation protocol is established. In some cases, these biomarkers may be present in very small quantities. The presence of an established SOP will minimise the effects of user variability during sample collection.

Disclaimer: *This protocol has been developed and tested for research use only, not for veterinary or clinical use. No claims are made for its usefulness, accuracy or safety, and no liability can be accepted for any damages, losses or other expenses of any nature whatsoever arising from its use or supply. This protocol does not cover any legal or ethical issues.*

3. Health and Safety

Handle all swab samples as a potential source of pathogens, use appropriate personal protective equipment and handle samples as potential carriers of (bio)hazardous materials. Users of this protocol should have read and understood all relevant Risk and COSHH Assessments and other relevant SOPs, in particular health and safety rules and regulations and SOPs for handling farm animals and their body fluids. Before attempting to use this SOP, it is important to read and understand this document in its entirety.

4. Recommended equipment/material and personnel required (per collection event)

4.1 Equipment

- Polystyrene transport box (35cm*35cm*35cm) filled with ice
- Black permanent marker
- Grip seal plastic bag
- Storage freezer box

4.2 Disposables

- Plain wood stick swabs with tube casing (Thermo Scientific™, F150CA SWA2999)

4.3 Personnel required

- Two trained research, farm or veterinary staff will be required for the whole duration of each sampling. This is necessary to ensure the safety of personnel and the safety and welfare of the animals being sampled.

5. Procedural guidelines

Only collect oral and nasal swabs whilst the animal is physically restrained in a cattle crush. The animal should then be haltered and tied to the side of the crush in such a way that the head cannot move. This is essential both for the safety of the staff taking the swabs and for the safety of the animal as there is a danger the swab could break off in nasal cavity or mouth/pharynx. Only experienced staff members should attempt to restrain a cow as inexperience leads to dangers for all involved.
Once the animal is secure within the crush and correctly haltered, the procedure of taking the swab can be followed.

5.1 Nasal Swab Sample Collection Procedure

5.1.1 Clean nostrils provide the best nasal samples. However, traces of dirt or feed in the animal's nostrils is acceptable though not desirable. Wherever possible, avoid collecting swab samples immediately after the animal has eaten. Ensure the collection end of the swab does not touch anything other than the animal's nostril to prevent contamination of the sample.

5.1.2 Holding the tube, unscrew the lid and carefully remove the swab. Immediately, rub the end containing the swab inside the animal's nostril for 3-5 seconds. Ensure the swab looks wet and is coated with the nasal sample to achieve best results.

5.1.3 Holding the tube in one hand, carefully insert the nasal swab into the tube. Screw cap on tightly to prevent any leakage during transport.

5.1.4 Using a permanent marker, clearly write the animal identification number, farm identification code and the type of swab (nasal or oral) on the side of the tube and place the tube in a grip seal bag and immediately on ice.

5.1.5 Go to 5.2 for oral swab sample collection.

5.2 Oral Swab Sample Collection Procedure

5.2.1 As before, avoid collecting swab samples immediately after the animal has eaten. Try to avoid traces of dirt or feed. Ensure the collection end of the swab does not touch anything other than the animal's mouth to prevent contamination of the sample.

5.2.2 Holding the tube, unscrew the lid and carefully remove the swab. Immediately, rub the end containing the swab on the inside base of the animal's mouth to coat in saliva for 5 seconds. Please note that too much pressure can result in swab breaking. Ensure the swab looks wet and is coated with the saliva sample to achieve best results.

5.2.3 Holding the tube in one hand, carefully insert the swab into the tube. Screw cap on tightly to prevent any leakage during transport.

5.2.4 Using a permanent marker, clearly write the animal identification number, farm identification code and the type of swab (nasal or oral) on the side of the tube and place the tube in a grip seal bag and immediately on ice.

5.3 Packaging for Transport

5.3.1 Following swab sample collection, the grip seal bags containing the swabs are placed into an ice-filled freezer transport box with the lid securely on for transportation.

5.4 Receipt of Samples in the Laboratory

5.4.1 Trained and vaccinated personnel will receive samples delivered to the Chemistry building and transport them straight to laboratory room 142 in the Chemistry building.

5.4.2 For samples to be stored, swabs will be stored in sample quarantine within a storage freezer box in a locked -80°C freezer in laboratory room 142 until required for sample processing.

5.4.3 For further processing, samples are moved into a microbiological safety cabinet where the packaging can be removed, making the sample accessible for downstream processing. Follow all relevant risk assessment for handling and further processing of the samples.

Document S2 – SOP for Nasal Swab Analysis by LAP-MALDI MS

Standard Operating Procedure (SOP) for nasal swab processing for the detection of disease in cattle by LAP-MALDI mass spectrometry analysis

Version 1.0 (March 2022)

Sophie Lellman and Rainer Cramer
Department of Chemistry, UoR

1. Purpose

The purpose of this document is to provide guidance for the processing of nasal swabs from cattle for analysis by liquid atmospheric pressure (LAP)-MALDI mass spectrometry (MS), and the subsequent data analysis and mining, for the detection of bovine tuberculosis (bTB).

2. Scope

This SOP has been designed for the appropriate processing of nasal swabs from cattle for the detection of bTB and potentially other diseases. Nasal swabs have been previously collected by trained research, farm or veterinary staff (see *Standard Operating Procedure (SOP) for oral and nasal swab collection for the diagnosis of bovine tuberculosis (bTB)* – version 1.3). The sample preparation protocol has been optimised to include a short digestion step to assist in the detection of biomarkers for disease classification. The following protocol is designed for data acquisition using LAP-MALDI MS and subsequent data analysis.

Disclaimer: *This protocol has been developed and tested for research use only, not for veterinary or clinical use. No claims are made for its usefulness, accuracy or safety, and no liability can be accepted for any damages, losses or other expenses of any nature whatsoever arising from its use or supply. This protocol does not cover any legal or ethical issues.*

3. Health and Safety

Swab samples to be used following this SOP will contain pathogens including causative agents of bTB (*Mycobacterium bovis*) and bovine mastitis (including but not limited to *Staphylococcus aureus, Streptococcus agalactiae, Escherichia coli* and *Streptococcus uberis*). *M. bovis* is a biosafety level 3 bacterium and therefore must be handled in a category 3 laboratory. A derogation from category 3 to category 2 for the handling of *M. bovis* containing samples can be applied for from HSE providing suitable health and safety measures. Bacterial species causing bovine mastitis are biosafety level 2 pathogens and can therefore be handled in a category 2 facility.
All swab samples, whether health status is known or not, should be handled as a potential source of pathogens, using appropriate personal protective equipment, and handled as potential carriers of (bio)hazardous materials. Users of this protocol should have read and understood all relevant Risk and COSHH Assessments and other relevant SOPs, in particular health and safety rules and regulations and SOPs for handling farm animals and their body fluids. Before attempting to use this SOP, it is important to read and understand this document in its entirety and ensure all suitable H&S procedures are in place before commencing.

4. Recommended equipment/material and personnel required

a. *Equipment*

- Bench-top centrifuge capable of 13,000 rpm with rotor for 1.5mL tubes
- Incubator oven set at 37°C
- Standard lab vortexer
- Synapt G2-Si mass spectrometer (Waters Corporation, Wilmslow, UK) with AP-MALDI source (detailed in Ryumin et al., 2016) and MALDI sample plate
- Microbiological safety cabinet (see section 3 for further details)

b. *Disposables*

- 1.5-mL tubes (Fisher Scientific, 05-408-129)
- C18 ZipTips (Merck, ZTC18S096)
- 0.1-10-µL pipette tips (Fisher Scientific, 0030073428)
- 2-200-µL pipette tips (Fisher Scientific, 00300734363)
- 5-1000-µL pipette tips (Fisher Scientific, 0030073460)

c. *Chemicals and Reagents*

- 1x Phosphate-Buffered Saline (PBS; prepared according to the 1X PBS recipe: https://www.sigmaaldrich.com/GB/en/support/calculators-and-apps/1x-phosphate-buffered-saline)
- Ethanol (Sigma-Aldrich, 32221-2.5L-M)
- Water, HPLC Grade (Fisher Scientific, W/0106/17)
- Acetonitrile (ACN), HPLC Grade (Honeywell, 608-001-00-3)
- Trifluoroacetic acid (TFA), LC-MS Grade (TFA) (Thermo Scientific, 85183)
- Ammonium bicarbonate (Sigma-Aldrich, 09830-500G) buffer, 50mM in HPLC-Grade H_2O
- Dithiothreitol (DTT) (Sigma-Aldrich, D0632-1G)
- Iodoacetamide (IAA) (Sigma-Aldrich, I61250-10G)
- Sequencing-grade modified Trypsin (Promega, V5111)
- α-Cyano-4-hydroxycinnamic acid (CHCA) (Sigma-Aldrich, 70990-250MG-F)
- Propylene glycol (PG) (Sigma-Aldrich, W294004-1KG-K)

d. *Personnel Required*

- One research scientist

5. Procedural guidelines

These procedures are for the preparation of bovine nasal swab samples collected as detailed in the *SOP for oral and nasal swab collection for the diagnosis of bovine tuberculosis (bTB)*,

v1.3. Swabs can be either directly analysed using this method, or thawed from quarantine in the -80°C freezer.

Note: It is advisable that all steps will be carried out by keeping the samples and extracts etc. on ice as much as possible. If storage time is needed between the various sample preparation stages, this should be minimized and kept the same within a (disease classification) study. For longer storage times, the samples should be kept in a -80°C freezer.

a. *Inactivation and Precipitation*

   i. Move the swabs to the microbiological safety cabinet (MSC), either from sample quarantine or directly from the transportation packaging.
   ii. Inside the MSC, carefully remove swab from its casing and place it into a 1.5-mL tube containing 400µL of 1X PBS, and briefly agitate (at least five times).
   iii. Discard swab after squeezing it gently against the inside walls of the 1.5-mL tube.
   iv. Add 900µL of 100% ethanol and vortex to mix. Samples should be left on ice if multiple samples are processed at the same time.
   v. Centrifuge samples for 5 minutes at 13,000 rpm. Remove and discard the supernatant and resuspend the resultant pellet in 30µL of 0.1% TFA.

b. *Trypsin Digestion*

   i. Add 50µL of 50mM ammonium bicarbonate to the samples and mix by pipetting.
   ii. Add 5µL of 100mM DTT and vortex. Incubate at 37°C for 30 minutes.
   iii. Add 10µL of 100mM IAA and vortex. Incubate at room temperature in the dark for 30 minutes.
   iv. Add 2µL of sequencing grade trypsin (0.2µg/µL) and incubate at 37°C for 2 hours.
   v. Add 8µL of 10% TFA to inactivate trypsin and stop the reaction.
   vi. Purify the samples and extract the peptides by using ZipTips according to the manufacturer's instructions, with a final elution volume of 5µL.

c. *Liquid MALDI MS Sample Preparation*

   i. Prepare a liquid support matrix (LSM) for MALDI analysis by dissolving CHCA in 70:30 ACN:H_2O at a concentration of 25mg/mL. The solution can be sonicated in an ultrasonic water bath to assist dissolving the CHCA crystals. Once fully dissolved, add PG (by adding 7 volume parts of PG to 10 volume parts of CHCA solution) and mix by vortexing.
   ii. For each sample, spot 0.5µL of the LSM on to a stainless steel MALDI sample plate followed by the addition of 0.5µL of the digest sample to the same spot.
   iii. Mount the MALDI sample plate on to the plate holder.

d. MS Data Acquisition

Note: The following steps and instrument settings are just for general guidance for liquid AP-MALDI MS analysis of samples as prepared in the previous steps on a Waters Synapt G2-Si instrument.

i. Use the following instrument modes: Mobility TOF, Positive Ion, Sensitivity and MS.
ii. Calibration of the instrument should be performed in Mobility TOF MS mode using sodium iodide over the m/z range of 100-2000.
iii. Set the laser energy to 18µJ/pulse and the laser pulse repetition frequency to 30Hz, continuous pulsing.

Note: The exact threshold laser energy that is best for an effective detection of analyte ion signals depends on the laser focus on the droplet surface and other ion source parameters (e.g. laser beam angle/position on droplet).

iv. Set the ion source to the following settings:
Temperature – 80°C;
Transfer Tube Voltage – 3.0kV;
Cone (counter) gas flow – 180L/h.
v. For data acquisition, use the following settings:
m/z range – 100-2000;
Scan Time – 1 minute;
Scans per Second – 1.

Details about automatic data acquisition and the associated sample stage movement depend on the exact plate size and analytes to be detected, amongst other ion source parameters and analytical objectives. Thus, no further details on automatic data acquisition will be given.

e. Data Analysis

For sample classification, there are many types of software that can be used for statistical modelling. In this case, we have used Abstract Model Builder (AMX, Waters). A training set of LAP-MALDI mass spectral profiles was generated for classification of healthy, mastitis and tuberculosis samples. Cross-validation of the model was performed in the absence of unknown samples to test against the model, whereby 20% of the training set data was left out and tested against the remaining data set.

Example -

i. Start the Abstract Model Builder (AMX) [Beta] version 1.01962.0 software.
ii. Switch to the 'Sample List' tab. Click 'Add sample files' and select all Waters .raw files to be added to the model, click on the single arrow tab and apply the correct classification to each file according to the sample type (healthy, mastitis, reactor).

iii. Click on ‘Add samples’, which takes you back to the ‘Sample list’ tab.
iv. Select all files and in the ‘TIC Operations’ toolbar, click on ’Spectrum from all scans’.
v. Switch to the ‘Model properties’ tab.
vi. Enter an appropriate model name in the ‘Model name’ field and select ‘LDA model’ from the ‘Model type’ drop-down menu.
vii. In the ‘PCA dimensions’ field, set the value to 10 (or less if you have less .raw files) and in the ‘LDA dimensions’ field, set the value to n-1 (n = number of classes). In the ‘Intensity limit’ field, enter 1.00E2.
viii. Binning is kept at default values, performed every 1 Da in the mass range of 650-2000 in advanced mode.
ix. Under ‘Spectrum interpretation’ select ‘One spectrum per burn’.
x. Under ‘Preprocessing’ only select ‘Apply background subtraction’ and ‘Apply normalization’.
xi. Switch to the ‘Model builder’ tab and click on ‘Build the model’. This can take up to 30 minutes, if a high number of data files are provided.
xii. Once the model building is complete, switch to the ‘Visualisation’ tab for a representation of the PCA/LDA model. By default, the first 3 components that are most responsible for variation are displayed. This can be altered by clicking the ‘…’ icon on the top toolbar, to the right of the text field, which indicates the displayed components.
xiii. Switch to the ‘Cross validation’ tab. Keep the default settings the same, which are the ‘20% out’ function, with the ‘Outlier’ drop down menu set to ‘Based on standard deviation’ and the ‘Std. dev. multiplier’ set to 5. Enter a suitable report name in the ‘New report name’ field and click on ‘Start’. This can also take up to 30 minutes.
xiv. Click ‘OK’ when the ‘Cross validation terminated successfully. The report can be seen on the reports page.’ window appears.
xv. Within the ‘Cross validation’ tab, switch to the ‘Reports’ tab. Select the report required, which can be identified by the name and creation time. Selecting the report will display the confusion matrix, the correct classification rate and the identification of outliers.
xvi. The cross validation report can be saved by clicking ‘Save…’ on the appropriate report. By default, this is saved as a .csv file. Click on ‘Save’ and subsequently on ‘OK’ when the ‘CSV file generated successfully.’ window appears.
xvii. To export the data matrix for use in other applications, switch to the ‘Home’ tab and select ‘Export data matrix’ in the top toolbar. This will export the data matrix according to the settings applied in the ‘Model properties’ tab as described in 5.5.6-5.5.10. This is exported as a .csv file.
xviii. When leaving the software, don’t forget to save the current project, following the prompts in the various windows that will appear.

6. References

Ryumin, P., et al. (2016). "Investigation and optimization of parameters affecting the multiply charged ion yield in AP-MALDI MS." Methods **104**: 11-20.

Figure S1 – LAP-MALDI Mass Spectrum of a bTB Sample (* Indicates the $[M+10H]^{10+}$ Signal of S100-A12)

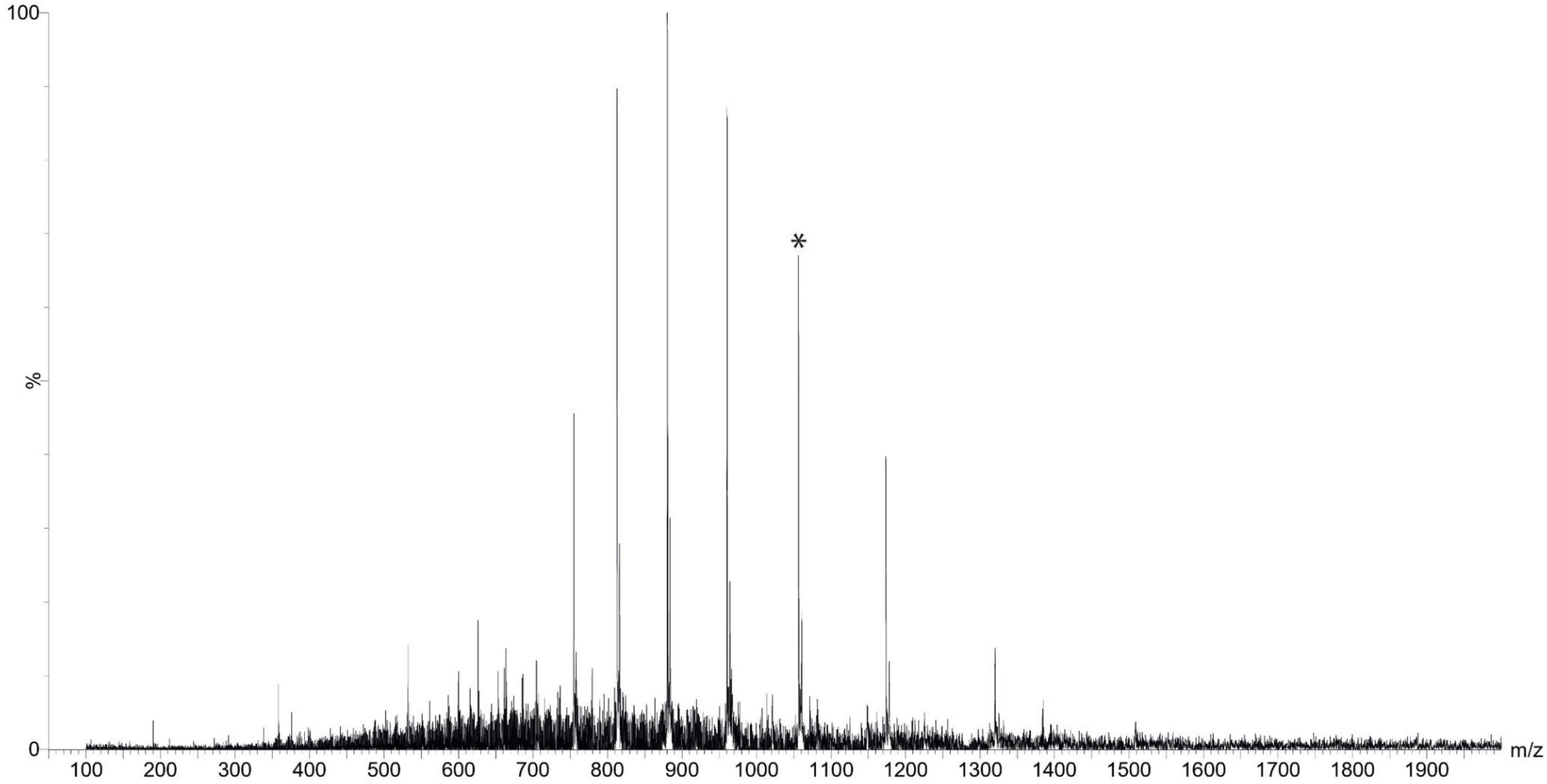

Figure S2 – Fragmentation Spectrum of the $[M+10H]^{10+}$ Signal of S100-A12

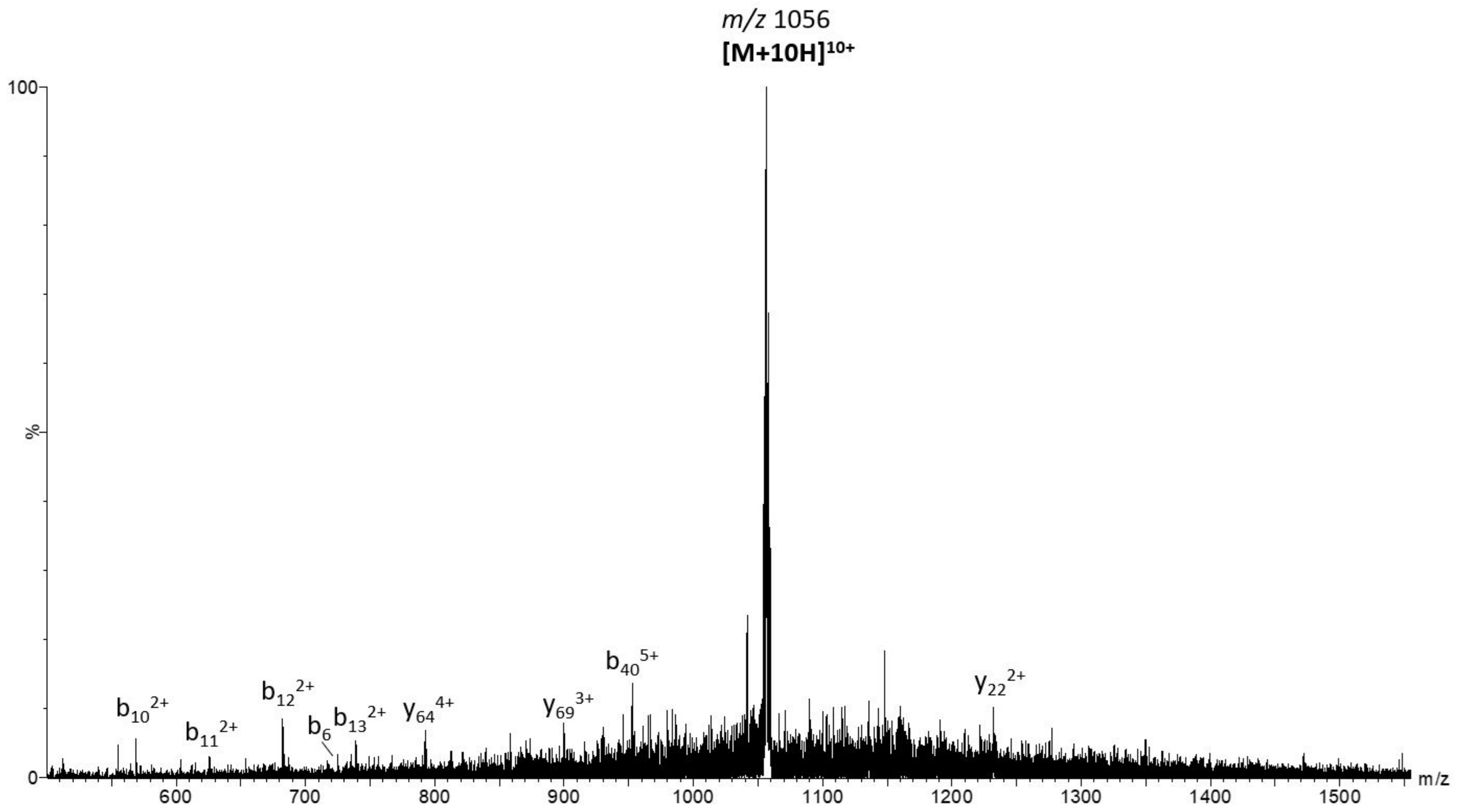

Document S3 – Peak List Used for Identifying S100-A12

BEGIN IONS

TITLE=Defra Reactor Protein Combined

PEPMASS=1319.4296

110.07 18749.00

120.08 12778.00

129.10 24703.00

266.16 3624.00

276.15 5837.00

366.18 3410.00

413.21 7690.00

467.22 2207.00

495.23 2389.00

587.30 4037.00

608.31 1324.00

632.33 623.00

724.37 3551.00

794.38 2257.00

1108.09 1728.00

1136.60 4687.00

1249.70 4147.00

3166.82 6347.00

1476.81 7343.00

3352.78 5065.00

1745.95 4596.00

1859.06 12400.00

4757.59 26190.00

1958.16 24310.00

5910.31 8428.00

4757.63 20180.00

END IONS
